# Response to symptoms of stroke in the UK: a systematic review

**DOI:** 10.1186/1472-6963-10-157

**Published:** 2010-06-08

**Authors:** Jan Lecouturier, Madeleine J Murtagh, Richard G Thomson, Gary A Ford, Martin White, Martin Eccles, Helen Rodgers

**Affiliations:** 1 Institute of Health and Society, Medical School, Newcastle University, Newcastle upon Tyne, UK; 2 Medical and Social Care Education, Leicester University, Leicester, UK; 3 Institute for Ageing and Health (Stroke Research Group), Medical School, Newcastle University, Newcastle upon Tyne, UK

## Abstract

**Background:**

The English National Stroke Strategy suggests that there is a need to improve the response of patients and witnesses to the symptoms of acute stroke to increase rapid access to specialist care. We wished to review the evidence base regarding the knowledge, attitudes and behaviours of stroke patients, witnesses and the public to the symptoms of stroke and the need for an urgent response at the onset of symptoms.

**Methods:**

We conducted a systematic review of UK articles reporting empirical research on a) awareness of and response to the symptoms of acute stroke or TIA, and b) beliefs and attitudes about diagnosis, early treatment and consequences of acute stroke or TIA. Nine electronic databases were searched using a robust search strategy. Citations and abstracts were screened independently by two reviewers. Data were extracted by two researchers independently using agreed criteria.

**Results:**

11 studies out of 7144 citations met the inclusion criteria. Methods of data collection included: postal survey (n = 2); interview survey (n = 6); review of hospital documentation (n = 2) and qualitative interviews (n = 1). Limited data reveal a good level of knowledge of the two commonest stroke symptoms (unilateral weakness and speech disturbance), and of the need for an emergency response among the general public and at risk patients. Despite this, less than half of patients recognised they had suffered a stroke. Symptom recognition did not reduce time to presentation. For the majority, the first point of contact for medical assistance was a general practitioner.

**Conclusions:**

There is an assumption that, in the UK, public knowledge of the symptoms of stroke and of the need for an emergency response is lacking, but there is little published research to support this. Public awareness raising campaigns to improve response to the symptoms of stroke therefore may not produce an increase in desired behaviours. Further research is needed to understand why people who experience or witness stroke symptoms frequently do not call emergency services.

## Background

Stroke is a major cause of death and disability world wide[1]. In England around 110,000 strokes and 20,000 TIAs occur each year and 300,000 people have been left with moderate to severe disabilities following a stroke[2]. As well as the personal cost of stroke to patients and their families, the estimated National Health Service annual expenditure in the UK on hospital and community care is approximately £2.8 billion[2]. Rapid access to a specialist service can reduce the risk of death and disability following stroke as early assessment enables accurate diagnosis, provision of acute treatment, early detection and response to complications, and provision of care by a multi-disciplinary team with expertise in stroke[3,4].

In 2007 the National Stroke Strategy was announced to provide a framework to promote stroke prevention and improve all aspects of stroke care in England[5]. The strategy outlines 20 quality markers with the first stating that 'members of the public and health and care staff are able to recognise and identify the main symptoms of stroke and know it needs to be treated as a medical emergency'[5]. The Department of Health subsequently launched a £12 million multi-media campaign in 2009 based on the FAST acronym (Face, Arm, Speech, Time to call emergency) to raise awareness of the three common symptoms of stroke and the need for an emergency response.

Early recognition and rapid response to the symptoms of stroke by patients and witnesses are important dimensions of improving access to thrombolysis and improving outcome following stroke. National Institute of Clinical Excellence recommend thrombolysis as an effective treatment for acute ischaemic stroke[6]. When given to carefully selected patients within three hours of the onset of symptoms intravenous tissue Plasminogen Activator (tPA) reduces the risk of death or dependency (odds ratio 0.64, 95% confidence intervals 0.5-0.83)[7]. If given within three hours one in three patients gain benefit from thrombolysis treatment and one in 33 are harmed by the treatment[8]. Recently published research suggests that thrombolysis may be beneficial up to 4.5 hours post stroke and this is likely to increase the number of patients eligible for this treatment[9]. Approximately 15% of patients are eligible for thrombolysis in the UK yet only 1% receive it[10] compared with 1.1% in the USA[11] and 3% in Germany[12]. Low thrombolysis treatment rates may be attributable to organisational issues or the knowledge or attitudes of health professionals but delay in presentation due to patient and public response to stroke symptoms may also be important. In 2002 the median time between onset of symptoms and arrival to hospital in the UK was six hours, with 37% arriving within three hours[13]. It is not clear whether patient and witness action at the onset of symptoms is related to a lack of knowledge or their beliefs and attitudes to stroke/TIA or its treatment.

We conducted a systematic review of UK studies to describe:

• patient and public awareness of the symptoms of stroke/TIA

• patient and public response to the symptoms of stroke/TIA

• the beliefs and attitudes of patients and the public about diagnosis, early treatment and consequences of acute stroke/TIA.

## Methods

Search terms were developed by the study team, then tested and adapted for each database. Table 1 describes the Medline search terms. We searched Medline (1980 to January 2010), CINAHL (1980-2010), EMBASE (1980-2010), CSA -, ASSIA, Sociological Abstracts - (1985-2010), PsychInfo (1980-2010), Web of Knowledge (1980-2010), ZETOC (1993-2010), AgeInfo (1980-2010) and the National Research Register (2000-2007).

**Table 1:** Medline search terms

**Patient and public awareness of the symptoms of stroke/TIA**	
1.	Stroke (mp) or CVA (exp) or TIA (mp) or ischaemic attack, transient or acute stroke (mp)
2.	Knowledge (mp) or knowledge (exp) or health knowledge, attitudes, practice (exp)
3.	Awareness (exp) or aware$ (mp)
4.	2 or 3
5.	Symptom$ (mp) or 'signs and symptoms' (exp)
6.	1 and 4 and 5.
	
**Patient and public response to the symptoms of stroke/TIA**	
7.	Health seeking behaviour (mp) or patient acceptance of health care (exp) or health knowledge, attitudes, practice (exp)
8.	Health service$ utlization (mp)
9.	Patient delay (mp)
10.	Health behaviour (exp) or health behaviour$ (mp)
11.	7 or 8 or 9 or 10
12.	1 and 11.
	
**Beliefs and attitudes of patients and the public about diagnosis, early treatment and consequences of acute stroke/TIA**	
13.	Attitude (mp) or attitude to health (exp) or attitude (exp)
14.	Beliefs (mp) or culture (exp)
15.	Public opinion (exp) or opinion$ (mp)
16.	View$ (mp)
17.	13 or 14 or 15 or 16
18.	Early diagnosis (exp) or oral, diagnosis (exp) or diagnosis(exp) or diagnosis (mp)
19.	Time factors (exp) or early treatment (mp)
20.	Consequences (mp)
21.	Fatal outcome (exp) or outcome$(mp)
22.	18 or 19 or 20 or 21
23.	1 and 17 and 22

### Inclusion and exclusion criteria

Articles reporting empirical research (using quantitative and qualitative methods) focusing on public and patient awareness of, and response to, the symptoms of stroke and TIA undertaken in the UK were included. Reviews and opinion pieces were excluded.

### Screening and data abstraction

Citations were initially screened on title and those retained were screened on abstract. This was done independently by JL and another researcher. Where there was insufficient information from the abstract to make a judgement, the full paper was obtained. Disagreements over the inclusion of studies were resolved by a third person (HR). The reference lists of key papers were searched to identify any further articles of relevance.

Two reviewers (JL, HR) independently reviewed the retained papers and extracted data into an ACCESS database. We developed a quality checklist to record: adequacy of measures to address research question; adequacy of sample size and method of sampling; representativeness of sample; response rate; and analysis of response bias. No exclusion criteria based upon the quality assessment were applied. Results are presented narratively as the studies identified were heterogeneous, used a range of designs, study populations and varied in the data items collected.

## Results

**Figure 1:**
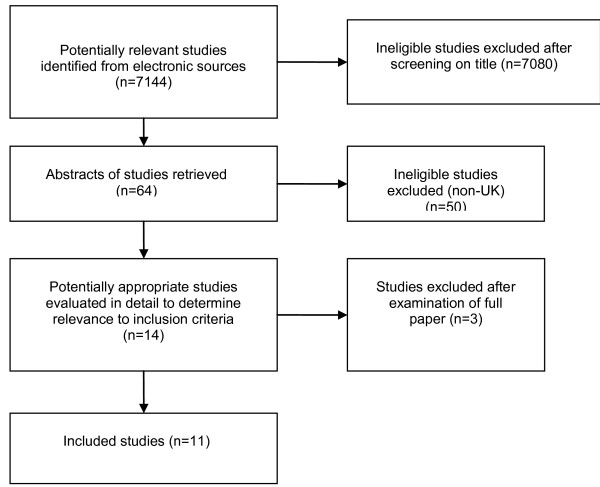
**Flow diagram of search results**.

The electronic search elicited 7144 citations of which 7131 were excluded after screening of title or abstract (Figure 1). Five relevant projects were identified from the National Research Register: three studies were ongoing, one had been completed but the results were not published (an abstract was obtained) and we had no response to our inquiries about the fifth. Fourteen publications were reviewed in full and 11 were eligible for inclusion in this review[13-23]. Table 2 provides a description of the included studies. Table 3 provides the main results from the included quantitative studies and illustrates how little published UK data is available.

**Table 2:** Description of included studies

First author, yr published,	Study type, data collection tool, year conducted	Study participants (n=)	Question(s) addressed			Recruitment
				
			Awareness of symptoms	Response to symptoms of stroke	Beliefs & attitudes	
Salisbury[15] 1998	Prospective cohort Structured interview schedule 1997	Stroke patients (177)	✓*	✓		New and recurrent stroke admissions to one hospital over a 6 month period.

Carroll[18] 2004	Survey	Stroke/TIA patients (40)	✓*	✓	✓	3 groups: patients with a diagnosis of stroke or TIA admitted within previous 48 hours; at risk patients attending hypertension, diabetic and chronic renal failure out-patient clinics; patients and relatives on non medical wards and visitors to the hospital café.
	Structured interview schedule & open ended questions	At risk patents (40)	✓		✓	
	2001/2	General public (40)	✓		✓	Response rates not given.

Townend[21] 2006	Mixed methods Structured interview & semi-structured interview 1 month after stroke & structured interview 9 months after stroke 2000/1	Stroke patients (89)			✓	Patients admitted to hospital with a diagnosis of stroke.

Giles[22] 2006	Survey Structured interview schedule 2002/3	TIA patients (241)	✓*	✓		2 cohorts of patients with TIA: one from a population-based study of the incidence of TIA and stroke (Oxford vascular study) and the other of patients referred to hospital TIA out-patient clinics recruited over a 12 month period.

Lasserson [14] 2008	Survey Structured interview schedule 2002/6	Minor stroke/TIA patients (768)		✓		A population based incidence study. Participants recruited from 9 general practices over a 4 year period (Oxford vascular study).

Shah[23] 2007	Survey Structured interview schedule 2002/3	Stroke patients & witnesses (103)	✓*	✓(patients only)	✓	Patients recently admitted with acute ischaemic stroke and witnesses.

Harraf[13] 2002	Observational study Structured proforma 2000	Stroke patients (739)		✓		Consecutive patients admitted to 11 teaching hospital and 11 district general hospitals with symptoms suggestive of an acute stroke over a 4 week period.

Harbison[17] 2004	Survey 2000	Stroke/TIA patients (487)		✓		The medical records of consecutive patients referred to a stroke service were prospectively studied over a six month period.

Parahoo[19] 2003	Postal survey 2001	General public (892)	✓		✓	Participants were randomly selected from electoral register.

Morgan[20] 2005	Postal survey Self-completion questionnaire 2003/4	General public (139)	✓		✓	Patients aged 40-65 were randomly selected from a general practice register. 57% response rate.

Gupta[16] 2002	Survey Structured interview schedule 1999	At risk patients (410)	✓			Patients attending a hospital clinic over an 8 month period with one or more established risk factors for stroke/TIA. Response rate not given.

*Patients were not tested on knowledge of symptoms but asked if they recognised they were having a stroke

**Table 3:** Main results of quantitative studies

			Patient and public awareness of the symptoms of stroke/TIA			Patient and public response to the symptoms of stroke/TIA				Beliefs and attitudes about the diagnosis, early treatment and consequences of stroke			
			**Associated with brain**	**Aware of one symptom**	**Recognised stroke/TIA**	**Time to seek medical help**	**Sought medical help as soon as possible**	**First medical contact GP**	**Patient or bystander called ambulance**	**Symptoms are serious**	**Always an emergency**	**Correctly identified 8 possible consequences**	**Stroke is risk factor for further stroke**

	**Author**	**n =**	**%**	**%**	**%**	**Median**	**%**	**%**	**%**	**%**	**%**	**%**	

**Public**	Parahoo[19]	892	60.3	92.2	-	-	-	-	-	-	-	8	-
	Morgan[20]	139	90	81	-	-	-	-	-	-	96	-	-
	Carrol[16]	40	-	87.5	-	-	-	-	-	-	92.3	-	-
	Shah[23]	103	-	-	65^a^	-	-	-	-	79^a^	-	-	-

**At risk**	Gupta[16]	410	86	82	-	-	-		-	-	-	-	5%
	Carrol[18]	40	-	92.5	-	-	-		-	-	86	-	5%

**Stroke/TIA patients**	Carrol[18]	40	-	-	40	30 min (max 6 days)	-	80	17.5	-	-	-	0%
	Giles[22]	241	-	-	42.2	-	44.4	86.7	-	-	-	-	-
	Lasserson [14]	768	-	-	-	-	-	75%^c^ 71%^d^	-	-	-	-	-

	Shah[23]	103	-	-	41	-	-	-	-	53	-	-	-
	Salisbury[15]	739	-	-	56^b^	15 min (max 5 days)	68	56	41	-	-	-	-
	Harraf[13]	739	-	-	-	-	-	50	43	-	-	-	-
	Harbison[17]	487	-	-	-	-	-	44	37	-	-	-	-

^a ^bystanders

^b ^patients or those with the patient at the onset of symptoms

^c ^TIA

^d ^Minor stroke

### Quality of included studies

The quality of reporting varied across studies: very little demographic data were provided and often the findings were reported without recourse to demographic variables. Response rates were not always given and it was not possible to examine response bias in the studies. Some sample sizes were small[14,18], study participants were not randomly or consecutively selected[16,18], studies were conducted in a small geographical area[18,20,16] or with a restricted group[20]. The one larger scale postal survey of the public conducted across Northern Ireland[19] reported that the level of knowledge they found may not reflect that of the wider population due to a disproportionate number of respondents to their survey with educational qualifications. In the two studies of at risk patients, one included only those over the age of 65 years[16] and whilst the other did not report any exclusions the mean age of participants was 68 years (SD 12.1)[18]. We found no published research on the level of awareness of younger people at risk of stroke. One study was published over 10 years ago and the behaviour of stroke patients, witnesses and professionals may have changed since then[15].

### Patient and public awareness of the symptoms of stroke or TIA

Four of the studies in this review examined level of awareness of the symptoms of stroke with the public and, or, at risk patients[16,18-20]. Knowledge was determined by asking participants to freely recall the symptoms[16,18] or to identify them from a list[19,20]. Symptoms of stroke were derived from or measured against the World Health Organisation Special Report on Stroke[16] and National Institute of Neurological Disorders[18] definitions (Additional File 1).

The majority of the public and patients at risk of stroke were aware of at least one stroke symptom: unilateral weakness and speech disturbance were the two symptoms of stroke most commonly mentioned or recognised. Younger respondents were more likely to be able to identify stroke symptoms[19,20]. Greater knowledge of stroke was not associated with gender, age or family history of stroke[19].

### Patient and public response to the symptoms of stroke or TIA

Seven studies collected data on health seeking behaviour following stroke [13,15,17,18,23] or TIA [14,22].

#### Stroke patients

Stroke patients or witnesses were more likely to contact a general practitioner than any other source of medical help[13,15,17]. The median delay in phoning for an ambulance or general practitioner was 15-30 minutes[15,18] and 79% sought help within one hour[15]. In another study, although the authors did not state actual times, 59% of affected patients waited to see if their symptoms resolved spontaneously compared to 25% of witnesses who waited[23]. Sixty one percent of patients and 80% of witnesses were concerned about bothering other people.

One study reported that the median time from onset of symptoms to arrival in hospital for those who used the emergency service was two hours and three minutes, and for those referred by their general practitioner it was seven hours and 12 minutes (odds ratio 0.45, 95% confidence intervals 0.23 to 0.61)[13]. A second study similarly reported that use of the emergency service reduced delays, as did onset not at home (p < 0.0001) and altered level of consciousness (p < 0.002)[15]. The median time from discovery to arrival at hospital was 2.63 hours for those who were in their own home compared with 1.6 hours in another's home or 0.8 hours in a public place. Those with the additional symptom of altered consciousness arrived in hospital a median of 1.5 hours: for those with vomiting it was 4.0 hours, for seizures 4.4 hours and headache 2.3 hours. Onset of symptoms between midnight and 6 a.m. was associated with delays greater than six hours between onset and arrival at hospital (odds ratio 1.22, 95% CI 1.04 to 1.45)[13].

#### TIA patients

In a study of TIA patients 44% did not seek medical attention for 24 hours: those with motor symptoms or those with symptoms lasting for more than 60 minutes were more likely to take emergency action[22]. There was no relationship between health seeking behaviour and age, sex, vascular territory of TIA or vascular risk factors, including previous stroke[22]. When a TIA or minor stroke occurred out of general practice surgery opening hours patients often delayed seeking medical attention until a member of their practice was available[22]. This was particularly an issue at weekends. Recognition of the symptoms of TIA did not influence whether or not patients sought immediate medical help or presentation time at hospital[14,22].

### Beliefs and attitudes of patients and the general public about diagnosis, early treatment and consequences of acute stroke or TIA

Seven studies focused on the beliefs and attitudes of patients and the general public about stroke[15,18-23].

#### Patient and witness recognition of stroke

Less than half of patients recognised they were having a stroke[15,23] or TIA[22] and some incorrectly attributed the symptoms to stress or fatigue (5%), eye problems (3%), migraine (1%) or heart attack (1%; 5%)[22,23]. TIA patients with motor symptoms were more likely to correctly interpret the symptoms than those without (49% vs 36% p = 0.046), as were those with previous TIA (58% vs 40% p = 0.044)[22]. At the onset of the stroke, witnesses were more likely than the patients to consider the symptoms as serious[23].

#### General public and at risk patients' views on early treatment

In two studies, with a total of 179 members of the public[20,18] and 40 patients at risk of stroke[18], the majority felt that stroke was always an emergency.

#### Views on consequences of stroke

Five per cent of at risk patients and none of the stroke patients interviewed were aware that suffering a stroke or TIA was a risk factor for further stroke[16]. One study, using a mix of quantitative and qualitative methods, explored stroke patients' views on the recurrence of stroke[21]. Fifty out of 89 participants were worried about having another stroke. The most common fear about recurrent stroke was severe disability resulting in lack of mobility and inability to communicate. Some patients expressed a fear of dying if they suffered a further stroke. These fears often stemmed from the experiences of friends or family members or what they witnessed in other patients in hospital[21]. Only one study explored public views on this issue and reported that the minority correctly identified all eight possible consequences[16]. Unfortunately the list of eight consequences was not given in the paper.

## Discussion

This review demonstrates that from the limited data identified there is a good level of knowledge of the two commonest stroke symptoms and of the need for an emergency response among the general public and at risk patients. There was a tendency for patients and witnesses to contact their general practitioner rather than call for an ambulance. Recognition that the symptoms were due stroke or TIA did not influence time to presentation. Very few studies examined the beliefs and attitudes of the public towards stroke and its treatment, factors that could be important in understanding why people do or do not respond to the symptoms of stroke as an emergency.

Although the public are most likely to recognise unilateral weakness and/or numbness and speech disturbance which are the most common symptoms of stroke, this conclusion is based upon data from 1071 participants in three studies and there are issues around the representativeness of the samples. The majority of the general public regard stroke as a serious condition requiring emergency treatment, based on a sample of only 179 respondents. Although less than half of stroke and TIA patients in the studies said they were aware they had suffered a stroke or TIA at the onset of symptoms, we do not know their level of knowledge about symptoms prior to the event. Cognitive impairment caused by the stroke may impact on the recognition and response to symptoms in some cases[22]. Surprisingly, the correct recognition of the symptoms of stroke or TIA did not influence whether or not initial help was sought via 999 or a general practitioner, and did not influence the time from onset of symptoms to arrival in hospital. Some patients and witnesses waited to see if symptoms resolved although it is not clear why this was the case. Factors such as denial and a reticence to bother others[23] - for example waiting until their local surgery was open before seeking medical attention[14] - may influence their decision about how, when and from whom to seek help. Stroke/TIA patients and witnesses were most likely to contact a general practitioner than any other source of medical help. Contact with a general practitioner significantly delayed the time between onset of symptoms and admission and will have reduced the number of patients who may have been eligible for thrombolysis if that service were available.

Studies from the USA and some parts of Europe report similar rates of awareness to the studies included in this review. For example, it is reported that 33-50% of stroke patients did not recognise that they had suffered a stroke[24] and delays in seeking appropriate medical care were associated with experiencing non-motor symptoms and not calling emergency services immediately[25]. In the USA the major delay in receiving emergency treatment following stroke is the time taken to seek medical care following the onset of symptoms[26]. There, and in other countries, this has been attributed to lack of awareness of stroke symptoms and waiting to see if symptoms resolve[24,26]. In relation to the general public, USA studies reported 69%[27] and 70%[28] could freely recall at least one stroke symptom and 89% correctly identified at least three major stroke symptoms from a list[29]. Similarly weakness and speech problems were the symptoms most commonly mentioned or recognised. Awareness was often poorest in those at a higher risk of stroke, for example older people and those from ethnic minorities[27,30].

### Limitations of study

We have systematically reviewed the literature on patient and public awareness of, and response to, the symptoms of stroke and TIA in the UK. The review focused on UK studies as findings from those conducted outside the UK of attitudes and behaviour are of limited value because of differences in culture and in the way healthcare systems are organised. Our search strategy was robust and relatively little published research was identified in this area. Several studies recruited a small number of participants from highly selected populations which in some cases were poorly described. In addition some studies excluded patients with communication difficulties. As a result these findings may not be generalisable to other settings. Most of the survey and interview studies reported response rates but none examined non-response. Awareness of, and response to, the symptoms of stroke may have improved since these papers were published, but this has not yet been reflected in a major increase in the number of patients who receive thrombolysis following ischaemic stroke[10], although this is likely to be influenced by other barriers to delivering thrombolysis. In addition, the 2005 National Audit Office Report (Reducing brain damage: faster access to better stroke care) concluded that an emergency response to stroke with efficient and effective acute care is generally lacking[2].

## Conclusions

Campaigns to increase awareness of stroke symptoms are based on the assumption that public knowledge is lacking about the common symptoms of stroke and the need for an emergency response. However, this review demonstrates that there is very little published data on how informed the UK public are about these issues. To minimise the time between onset of symptoms and access to specialist care it is necessary to understand the factors which influence how and when patients and witnesses seek help following a stroke. These factors may be demographic, social, cultural, behavioural, and perceptual or may relate to the presenting symptoms; those which influence the response of stroke patients and witnesses may differ. Our findings reinforce the need for robust studies to provide a better understanding of current awareness of stroke symptoms and the response of patients and the public. This will aid the development of strategies to improve emergency response to stroke.

## Competing interests

GF chaired the English Stroke Strategy Emergency Response group and is a member of the Department of Health Stroke Improvement Programme Board. GF has received honoraria for consulting and educational activities, and his Institution has received grants, from Boehringer Ingelheim Ltd.

## Authors' contributions

All authors were involved in the development of the search strategies. JL conducted the searches and electronic sifting of titles and abstracts. JL and HR independently reviewed the retained papers and extracted data. JL and HR wrote the first draft of the manuscript. All authors commented on the first draft and all revisions. All authors read and approved the final manuscript.

## Pre-publication history

The pre-publication history for this paper can be accessed here:

http://www.biomedcentral.com/1472-6963/10/157/prepub

## Supplementary Material

Additional file 1 **Measures of stroke symptoms**. Outlines two established lists of stroke symptoms some studies used to measure respondent knowledge against. Click here for file
