# Supplementary material for: Response to symptoms of stroke in the UK: a systematic review
Source: BMC Health Serv Res. 2010 Jun 8;10:157. doi: 10.1186/1472-6963-10-157 (PMC2911429; doi:10.1186/1472-6963-10-157)
Supplement: Additional file 1 — Measures of stroke symptoms. Outlines two established lists of stroke symptoms some studies used to measure respondent knowledge against. [file 1472-6963-10-157-S1.DOC]

**Figure 2 . Measures of stroke symptoms**

| World Health Organisation Special Report on Stroke | |
| --- | --- |
|  | sudden one-sided weakness/paralysis |
|  | loss of vision |
|  | loss of speech |
|  | inability to walk |
|  | sudden numbness |
| National Institute of Neurological Disorders | |
|  | sudden numbness or weakness of the face, arm, or leg, especially on one side  of the body |
|  | sudden confusion, trouble talking, or understanding speech |
|  | sudden trouble seeing in one or both eyes |
|  | sudden trouble walking, dizziness, or loss of balance or coordination |
|  | sudden severe headache with no known cause |
